# Drug-Release Mechanisms Elucidated by Imaging Techniques: Visualizing the Invisible!

**DOI:** 10.3390/pharmaceutics14061165

**Published:** 2022-05-30

**Authors:** Susanne Florin-Muschert

**Affiliations:** Univ. Lille, Inserm, CHU Lille, U1008, F-59000 Lille, France; susanne.muschert@univ-lille.fr

Imaging techniques such as Raman spectroscopy, electron microscopy, laser scanning confocal microscopy, atomic force microscopy, tomography, magnetic resonance imaging, and terahertz are powerful tools to elucidate drug-release mechanisms from different types of delivery devices.

Drug penetration, as well as mass transport mechanisms, can be highly complex, and applying theoretical approaches to interpret underlying mass transport mechanisms might cause misguidance, especially if one of the conditions (of the prediction model) involuntary changes during the dissolution test [1].

Images offer the advantage of picturing the fate of the active molecule within the dosage form or at the site of action and give highly useful supplementary information during dissolution tests.

This Special Issue collected high-standard original research articles on the application of imaging techniques to explain dug delivery (Figure 1). There were seven papers submitted, and five of them have been published.

Kaushik et al. reported very interesting findings on the penetration mechanisms of a dye from Vaseline-based ointment [2]. Thanks to inverted epifluorescence microscopy, the authors showed that there are three mechanisms of penetration of the active ingredient into the skin, (i) by convection or solvent-dragged penetration, (ii) via passive diffusion of the active ingredient, and (iii) local penetration phenomena (for example, liquid menisci due to massage).

A capsule-in-capsule drug delivery device was analyzed in terms of the disintegration time and location as a function of the capsule material [3]. The MRI images of black iron oxide (in the inner capsule) revealed capsule disintegration in different regions of the gastrointestinal (GI) tract and have been compared to the established method of tracing salivary caffeine (radio-labeled in the inner capsule and natural caffeine in the outer capsule). Unfortunately, hibiscus tea powder also placed in the outer capsules could not be visualized upon capsule disintegration via the MRI method. Rump et al. showed the reliability of the two analyzing techniques to monitor the fast disintegration time for single capsules and the delayed disintegration time for the capsule combinations. Here, inter-subject GI variability caused variance in the disintegration time, which was higher with delayed liberation of capsule content.

Dong and co-workers investigated water transport into immediate-release tablets of varying formulations and microstructures [4]. With these types of tablets, water transport is of utmost importance to control drug release and tablet disintegration. Terahertz pulsed imaging allows monitoring the advancement of the waterfront within the tablet upon contact with the dissolution medium. Interestingly, tablet porosity predominantly impacted tablet dissolution.

Fahier et al. used Raman spectroscopy to monitor changes in core and film coating composition of pellets loaded with the weakly basic drug verapamil HCl and coated with a polymer blend [5]. The confocal Raman microscopic images allowed for non-invasive monitoring of pore creation within the film coating due to leaching of the water-soluble polymer. Fluorescence spectroscopy was also used to trace changes in the pH within the coated single pellets non-invasively.

Bollmann and Kleinebudde used X-Ray micro-computed tomography images of real pharmaceutical tablets to design virtual matrices (such as cylindrical tablets) and predicted the dissolution of the latter [6]. Thus, the ratio of active ingredient (API) and polymer in the binary mixtures, as well as the porosity of the matrix, could be virtually varied, and the dissolution profile could be calculated. The drug-release mechanism was either purely diffusion-controlled for lower API contents, or erosion of the matrices occurred with higher API contents. Thus, in silico predictions were based on the Higuchi and Korsmeyer-Peppas plot. However, with pharmaceutical systems with diffusion-based drug-release mechanisms, the dissolution could not be predicted in silico. This work adds important considerations to the long journey of in silico formulation development.

This Special Issue offers a selection of possibilities to employ imaging techniques to explain drug mobility and the disintegration of pharmaceutical dosage forms. This collection of high-quality scientific articles shows the diversity of application areas and the multitude of information that can be drawn from this type of analytical tool.

## Figures and Tables

**Figure 1:**
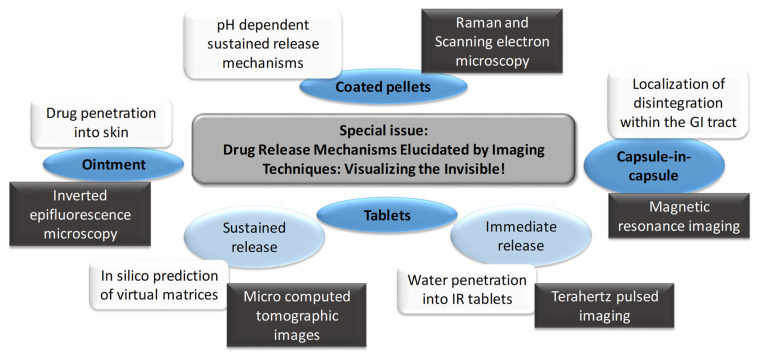
Schematic presentation of the Special Issue’s contents.
